# Clinical significance of *PCDH10* promoter methylation in diffuse large B-cell lymphoma

**DOI:** 10.1186/s12885-017-3810-7

**Published:** 2017-12-04

**Authors:** Wenting Huang, Xuemin Xue, Ling Shan, Tian Qiu, Lei Guo, Jianming Ying, Ning Lu

**Affiliations:** Department of Pathology, National Cancer Center/Cancer Hospital, Chinese Academy of Medical Sciences and Peking Union Medical College, Beijing, 100021 China

**Keywords:** PCDH10, Diffuse large B-cell lymphoma, Methylation, Prognosis

## Abstract

**Background:**

*PCDH10*, one of the non-clustered protocadherins, is identified as a tumor suppressor gene in many tumors. Recently, promoter methylation of *PCDH10* was found in diffuse large B-cell lymphoma (DLBCL) but not in normal lymph nodes, suggesting that its epigenetic aberrance is essential to the lymphomagenesis. However, there are few studies on the clinicopathological relevance and prognostic significance of PCDH10 methylation status in DLBCL.

**Methods:**

One hundred-seven cases of DLBCL between Jan 2009 and Jul 2010 were selected to extract genomic DNA and perform bisulfite modification. Their methylation status of *PCDH10* promoter were accessed by methylation-specific PCR (MSP) with methylated and unmethylated primers. Analysis of overall survival and clinicopathological correlation were conducted.

**Results:**

PCDH10 hypermethylation were found in 54.2% (58/107) of DLBCL cases, but only 12.5% (1/8) in reactive lymph node/follicular hyperplasia. In RCHOP-treated cohort, promoter methylation of PCDH10 is an independent prognostic indicator of worse overall survival (*p* = 0.017; HR 4.045; 95%CI 1.287–12.711) and worse progress-free survival (*p* = 0.014; HR 2.977; 95%CI 1.245–7.119). Whereas, PCDH10 hypermethylation wasn’t correlated with MYC translocation and cell of origin classification using Hans model.

**Conclusions:**

*PCDH10* methylation status could serve as a valuable biomarker for risk classification, and a potential therapeutic target for demethylating drugs in DLBCL in the future.

**Electronic supplementary material:**

The online version of this article (10.1186/s12885-017-3810-7) contains supplementary material, which is available to authorized users.

## Background

*PCDH10* is one of the non-clustered protocadherins encoding calcium-dependent adhesion protein, participating in multiple molecular functions, such as cell adhesion, colony formation and signaling regulation [1, 2]. It was identified as a tumor suppressor gene in many tumors, including nasopharyngeal carcinoma [2], gastric carcinoma [3] and multiple myeloma [4]. Epigenetic disruption of *PCDH10* was proved to be the key event leading to the transcriptional silencing or reduction [2]. Recent studies have shown that the methylation of *PCDH10* promoter could serve as a prognostic marker in gastric carcinoma [3, 5] and non-small-cell lung cancer [6].

Diffuse large B-cell lymphoma (DLBCL) is a molecular heterogeneity disease with wide spectrum of survival. Compared to the conventional CHOP chemotherapy, the prognosis of patients has been significantly improved by the addition of Rituximab, however there are still ~35% of DLBCL that are poor response [7]. International Prognostic Index (IPI) is confirmed as a robust prognostic indicator [8] but with little insight into the molecular mechanism. The cell of origin (COO) classification based on gene expression profiling shows great values of prognostic stratification [9] and clinical therapy selection [10], but it cannot be applied to the routine practice due to technical obstacles. Recently, silence or reduction of *PCDH10* and its promoter methylation was found in 80%(16/20) of DLBCL samples but not in the normal lymph nodes, suggesting that epigenetic aberrance of *PCDH10* is essential to the lymphomagenesis [11]. However, there are few studies on its clinicopathological relevance. Herein, our study will explore the relationship between *PCDH10* methylation status and prognostic significance in our cohort of DLBCL.

## Methods

### Study population

One hundred and seven cases of DLBCL with formalin-fixed, paraffin-embedded (FFPE) tissue blocks, were enrolled into this study between Jan 2009 and Jul 2010. All patients were diagnosed at National Cancer Center/Cancer Hospital, Chinese Academy of Medical Sciences according to the 4th edition of the *WHO Classification of Tumours of Haematopoietic and Lymphoid Tissues.* Treatment and prognosis data were collected using medical records review and telephone survey. Patients who received resection plus RCHOP or RCHOP alone were included in the survival analysis. Eight cases of reactive lymph node/follicular hyperplasia (RL/FH), six cases of chronic tonsillitis and nine cases of Castleman disease were selected as controls.

### Methylation-specific PCR(MSP)

Genomic DNA were extracted from FFPE blocks using QIAamp DNA FFPE Tissue Kit (Qiagen, Valencia, CA), and then bisulfite-treated using EZ DNA Methylation-Gold™ Kit (Zymo Research, Irvine, CA). The MSP primers were used as previously described, including methylated forward primer (5′-TCGTTAAATAGATACGTTACGC-3′), methylated reverse primer (5′-TAAAAACTAAAAACTTTCCGCG-3′), unmethylated forward primer (5′-GTTGTTAAATAGATATGTTATGT-3′), and unmethylated reverse primer (5′-CTAAAAACTAAAAACTTTCCACA-3′) [11]. MSP was performed using standard conditions for 40 cycles. The annealing temperatures of methylation and unmethylation primers were 60 °C and 58 °C, respectively. The product lengths of methylation and unmethylation PCR were 153 bps and 156 bps, respectively. The MSP products were checked on 2% the Agarose GEL. The methylated or unmethylated result was determined according to the band produced by which primers. In order to confirm the gel results, 2 of the methylation-positive PCR products were randomly selected to perform Sanger sequencing.

### Statistical analysis

The association between *PCDH10* methylation status and other clinicopathological characteristics was analyzed using Chi-square test and Fisher exact test. Overall survival (OS) is defined as time from the date of pathologic diagnosis to date of death from any cause. Progress-free survival (PFS) is defined as time from the date of pathologic diagnosis to the date of the progression or death, whichever occurs first. Univariate analysis for each parameter of interests is performed using Cox proportional hazard model. Parameters with *p*-value <0.05 from the univariate analysis are included in the multi-variate analysis using Cox PH regression model. Kaplan-Meier method is used to estimate the survival rate and plot the survival curve. The cutoff date was Jan 1st 2014. All statistical tests were two sided with *p* < 0.05 as significance. All statistical analyses were conducted in the IBM SPSS Statistics v22.

## Results

### Patients’ characteristics

The median age of our DLBCL cohort was 55 years (range19–88 years), about 54% of them were male. Primary nodal lymphoma accounted for 57.94% of the total cases. The proportions of the 4 IPI stratification groups from low to high risk were 44.86%, 28.04%, 18.69% and 8.41% respectively. According to the Hans algorithm, 30.8% cases were classified into GCB subtype. 8.41% patients were found with *MYC* translocation.

### Frequency of *PCDH10* promoter methylation in DLBCL

**Fig. 1:**
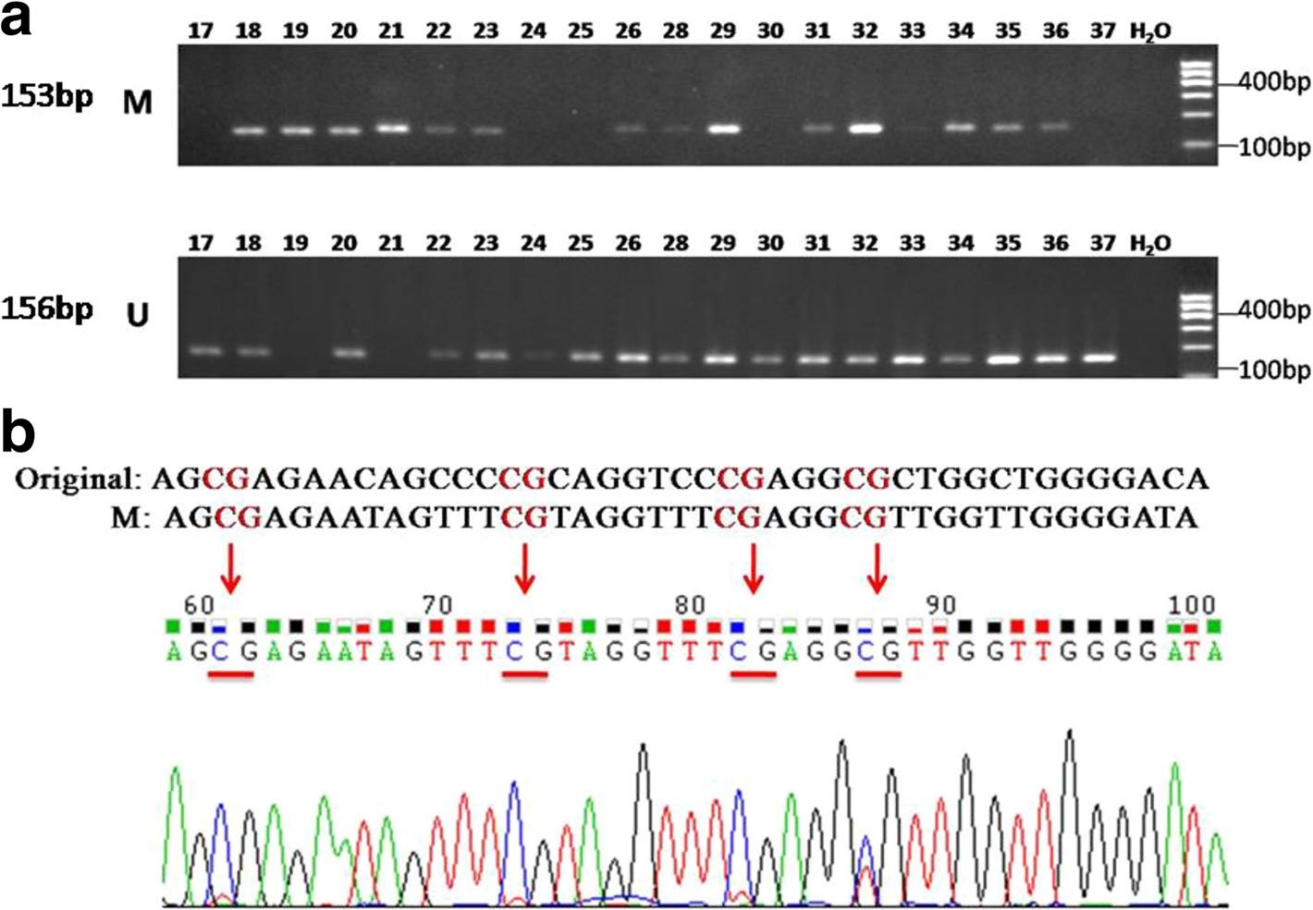
Methylation analysis of PCDH10 promoter in DLBCL. **a** Representative results of *PCDH10* MSP products using agarose GEL. *M*, methylated *PCDH10* promoter (PCR product length was 153 bp); *U*, unmethylated *PCDH10* promoter (PCR product length was 156 bp). **b** MSP products of methylated *PCDH10* promoter were confirmed by Sanger sequencing

One hundred-seven cases of DLBCL were analyzed using MSP, and 58 (54.2%) cases were found with *PCDH10* promoter methylation (Fig. 1). The specificity of MSP was confirmed by Sanger sequencing (Fig. 1). In contrast, only 12.5% (1/8) RL/FH were found with PCDH10 methylation, and all of them demonstrated polyclonal results of IgH and TCR by clonality analysis. Among the 9 cases of Castleman diseases, only 1 was found PCDH10 methylation, and no methylation was detected in the 6 cases of chronic tonsillitis.

### Correlation of *PCDH10* methylation and clinicopathological characteristics

The primary nodal presentation occupied 51.72% in DLBCL with *PCDH10* methylation, which was less than 65.31% in the patients without methylation. However, the correlation analysis by chi-square test didn’t show any significance (*p* = 0.156). The proportion of GCB subtype in methylation and non-methylation groups were 24.14% and 38.78% respectively(*p* = 0.102). The *MYC* translocation in methylation group (5.17%) was less than that in non-methylation counterpart (12.24%), while there wasn’t significantly different. Similarly, no statistical correlation of *PCDH10* methylation status with age (*p* = 0.282), sex (*p* = 0.575) and IPI risk category (*p* = 0.683) were obtained in our cohort (Table 1 and Additional file 1: Table S1).

**Table 1:** Clinicopathological characters and PCDH10 methylation status

	*PCDH10* Methylation		*p* value
	Yes (*n* = 58)	No (*n* = 49)	
Age at diagnosis			
<60y	39	28	0.282
≥ 60y	19	21	
Sex			
Male	30	28	0.575
Female	28	21	
Primary site			
Extranodal	28	17	0.156
Nodal^a^	30	32	
IPI risk category			
Low	26	22	0.683
Low-intermediate	15	15	
High-intermediate	13	7	
High	4	5	
Hans Algorithm			
GCB	14	19	0.102
Non-GCB	44	30	
*MYC* FISH Breakapart			
No	55	43	0.296^a^
Yes	3	6	

^a^Fisher test

### Survival analysis of DLBCL with RCHOP treatment

Survival analysis was performed in 65 cases of patients who received RCHOP regiment with or without surgical resection. The median follow-up was 58.7 months (range 3.0–82.7 months).

**Fig. 2:**
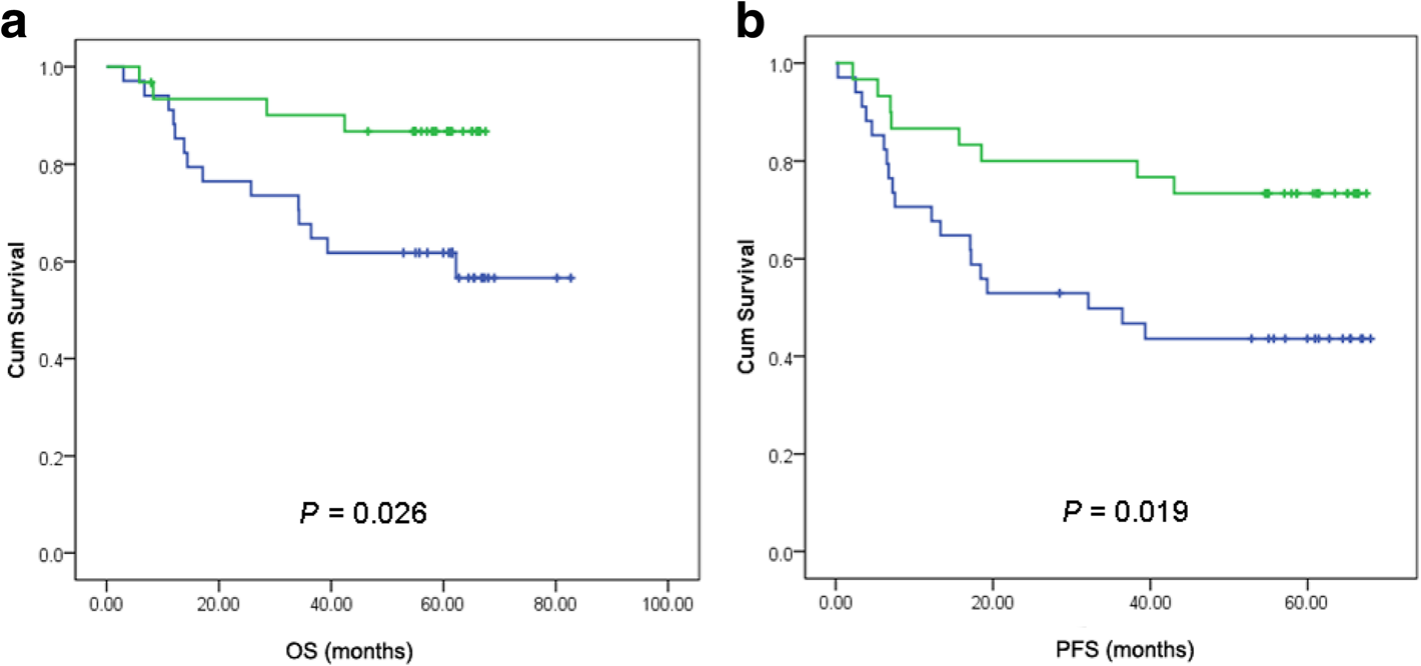
Survival curves of OS (**a**) and PFS (**b**) separated by *PCDH10* promoter methylation status. Blue and green line indicate PCDH10 with and without methylation, respectively. The *p*-values were calculated based on univariate Cox proportional regression analysis

Patients with methylation of *PCDH10* promoter were more likely to have dismal survival. The 3-year OS rate of patients with and without *PCDH10* methylation were 61.8% and 86.8% respectively (*p* = 0.026, Fig. 2). The 3-year PFS rate was 43.6% in patients with *PCDH10* methylation, as compared to 73.3% without methylation (*p* = 0.019, Fig. 2).

In the univariate survival analysis, IPI risk category and *PCDH10* methylation status were significantly associated with OS, and both of them were proved to be independent prognostic indicators by multivariate analysis (Table 2). With regard to PFS, three parameters, including IPI risk category, *PCDH10* methylation status and treatment, were found statistical significance using univariate analysis, and all of them were identified as independent risk predictors by multivariate analysis (Table 3 and Additional file 2: Table S2).

**Table 2:** Survival analysis of OS

	OS			
	HR_U(95%CI)	*p* value	HR_M(95%CI)	*p* value
Age at diagnosis				
<60y	1.000			
≥ 60y	1.393(0.549–3.535)	0.485		
Sex				
Male	1.000			
Female	0.426(0.160–1.137)	0.088		
Primary site				
Extranodal	1.000.			
Nodal^a^	2.267(0.744-6.911)	0.150		
IPI risk category				
Low	1.000		1.000	
Low-intermediate	2.029(0.545–7.559)	0.292	2.510(0.669–9.421)	0.173
High-intermediate	3.385(0.976–11.746)	0.055	2.836(0.815–9.873)	0.101
High	5.487(1.467–20.517)	0.011	6.986(1.845–26.450)	0.004
Treatment				
RCHOP	1.000			
Resection & RCHOP	0.152(0.020–1.145)	0.067		
Hans Algorithm				
GCB	1.000			
Non-GCB	1.419(0.505–3.984)	0.506		
*PCDH10* Methylation				
No	1.000		1.000	
Yes	3.547(1.166–10.791)	0.026	4.045(1.287–12.711)	0.017
*MYC* FISH Breakapart				
No	1.000			
Yes	1.285(0.170–9.713)	0.808		

*HR_U* hazard ratio by univariate analysis, *HR_M* hazard ratio by multivariate analysis

^a^DLBCL arising in spleen was considered as primary nodal lymphoma [20]

**Table 3:** Survival analysis of PFS

	PFS			
	HR_U(95%CI)	*p* value	HR_M(95%CI)	*p* value
Age at diagnosis				
<60y	1.000			
≥ 60y	1.557(0.731–3.314)	0.251		
Sex				
Male	1.000			
Female	1.067(0.501–2.272)	0.867		
Primary site				
Extranodal	1.000			
Nodal^a^	2.422(0.976-6.007)	0.056		
IPI risk category				
Low	1.000		1.000	
Low-intermediate	2.456(0.825–7.313)	0.107	2.474(0.829–7.389)	0.105
High-intermediate	3.895(1.360–11.151)	**0.011**	3.687(1.259–10.795)	**0.017**
High	9.202(3.156–26.832)	**<0.001**	8.680(2.874–26.215)	**<0.001**
Treatment				
RCHOP	1.000		1.000	
Resection & RCHOP	0.090(0.012–0.668)	**0.018**	0.119(0.016–0.903)	**0.040**
Hans Algorithm				
GCB	1.000			
Non-GCB	1.410(0.617–3.222)	0.415		
PCDH10 Methylation				
No	1.000		1.000	
Yes	2.687(1.173–6.156)	**0.019**	2.977(1.245–7.119)	**0.014**
*MYC* FISH Breakapart				
No	1.000			
Yes	0.716(0.097–5.279)	0.743		

*HR_U* hazard ratio by univariate analysis, *HR_M* hazard ratio by multivariate analysis

^a^DLBCL arising in spleen was considered as primary nodal lymphoma [20]

**Fig. 3:**
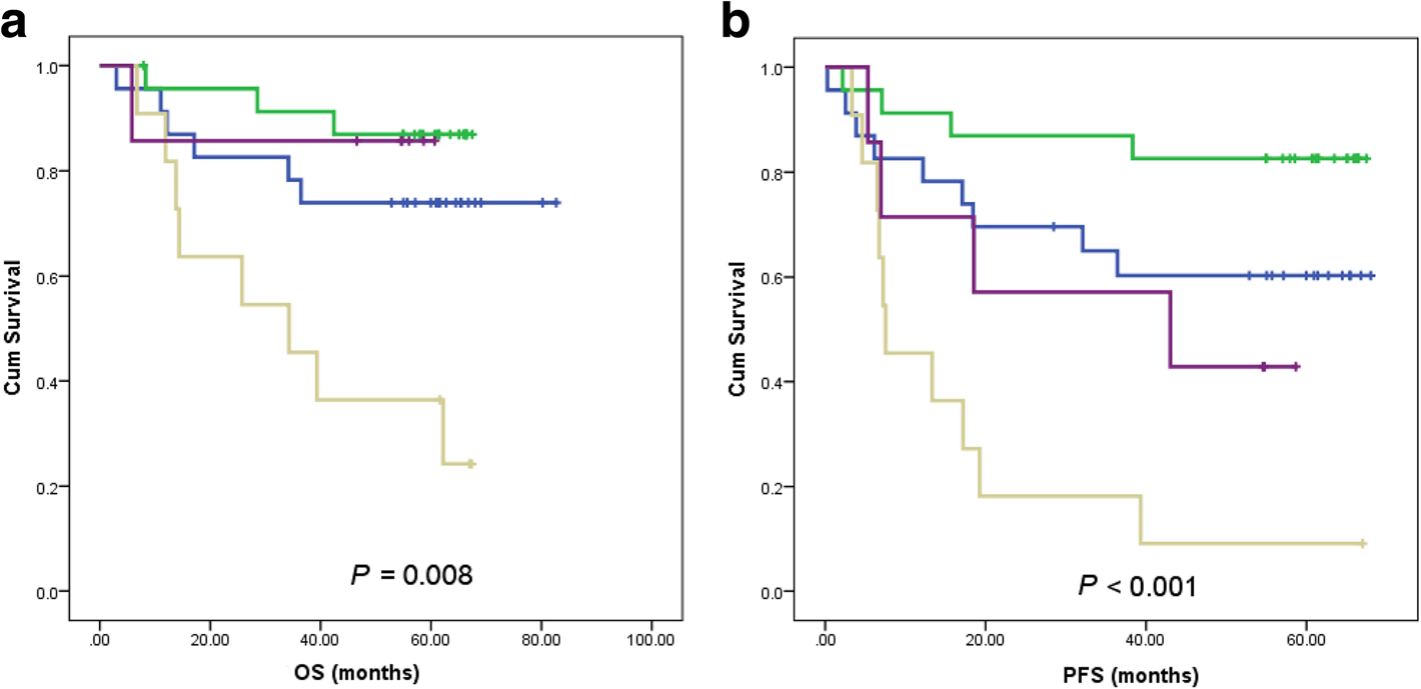
Survival curves of OS (**a**) and PFS (**b**) separated by the combination of IPI risk category and *PCDH10* methylation status. The blue line indicates IPI (1&2) plus PCDH10 with methylation, the green line indicates IPI (1&2) plus PCDH10 without methylation, the brown line indicates IPI (3&4) plus PCDH10 with methylation and the purple line indicates IPI (3&4) plus PCDH10 without methylation

Since the IPI risk category and *PCDH10* methylation were independent factors in both OS and PFS, we combined these two elements to establish a risk stratification. According to the log-rank analysis, the survival curves are greatly separated, and the *p* values of OS and PFS were 0.002 and <0.001, respectively. The survival of *PCDH10* methylation DLBCL was worse than that of non-methylation counterpart in both IPI (1&2) and IPI (3&4) groups (Fig. 3).

## Discussion

DLBCL is a highly heterogeneous lymphoma at multiple genetic levels. Gene expression profiling identified two kinds of classifications: the cell of origin classification (GCB, ABC, TypeIII) [12] and consensus cluster classification (BCR, OxPhos, HR) [13]. Recently, genome-wide DNA methylation study based on HELP microarray revealed a distinctive epigenetic classification, in which 6 groups (A to F groups) can be categorized and many protocadherins were found to be hypermethylated [14].

*PCDH10* is one of non-clustered protocadherins that belong to delta subfamily [1]. Its frequent epigenetic inactivation was reported in DLBCL [11]. In Chambwe et al. study, *PCDH10* were hypermethylation in 3 out of 6 epigenetic clusters, occupying approximately 34.3% of the whole cohort [14]. And an even higher frequency (100%) in Narayan et al. study was reported by using MSP [15]. Coupled with our data of 54.2% methylation rate, *PCDH10* promoter methylation could be a common event and may play an important role in lymphomagenesis. The frequency of Narayan et al. study seems to be higher than that of our study, probably because the coverage varied among those primers (The detail of coverage of our primers can be found in Additional file 3: Diagram S1).

Our study also examined the RL/FH, chronic tonsillitis and Castleman diseases, and found that the frequency of *PCDH10* methylation were 12.5%, 0% and 11.1% respectively. The incidence of methylation was much lower than that of DLBCL, implying that *PCDH10* promoter methylation is a characteristic of lymphoid malignancy. This result is consistent with the findings of Narayan et al. study [15].

DNA epigenetic disruption could exert crucial effects on the gene expression. Recent research has revealed several distinct signatures of DNA methylation between GCB and ABC groups of DLBCL [16]. Our study also explored the relationship between *PCDH10* methylation and COO classification based on Hans algorithm. No statistical significance was achieved (*p* = 0.102). This result was in line with the current study using HELP microarray. The data showed that three clusters (C/E/F clusters) with *PCDH10* hypermethylation didn’t show any preference to ABC or GCB subtypes, as compared with the rest *PCDH10*-unmethylated clusters (A/B/D clusters) [14]. Similarly, no significant correlations were found between *PCDH10* promoter methylation and other clinicopathological parameters, including the IPI risk category and *MYC* breakapart.

Furthermore, the prognostic prediction of *PCDH10* methylation revealed in many carcinomas was also investigated in our RCHOP-treated cohort [3, 5, 6]. Similar to IPI risk category, *PCDH10* methylation status was identified as an independent prognostic parameter for OS and PFS. Patient with *PCDH10* methylation showed more aggressive clinical behavior. Unfortunately, the detailed mechanism remained unclear. Narayan et al. study showed that B-NHL cell lines harbored *PCDH10* promoter methylation were less sensitive to doxorubicin treatment. And the cell lines with homozygous methylation showed less cytotoxicity as compared with that of heterozygous methylation [15]. These results may explain the short OS and PFS observed in our *PCDH10*-methylated patients, since doxorubicin is also an important part of R-CHOP regime.

The tumorigenesis mechanisms of *PCDH10* were most studied in the multiple myeloma(MM), while little is known in DLBCL. In MM, *PCDH10* silencing was reported that can enhance migration of b-catenin to nucleus, forming the complex of b-catenin/LEF/TCF and consequently promoting the MM cell growth [4]. In DLBCL, the aberrance of Wnt/b-catenin signaling also played an essential role in the pathogenesis [17]. The nuclear localization of b-catenin was found in nearly half of lymphoma, and the mRNA and protein levels of b-catenin were higher than that of lymph nodes [18]. Herein, we postulated that methylated *PCDH10* may promote lymphomagenesis by means of Wnt/b-catenin signaling. Another most likely mechanism involved was NF-κB pathway. Current study in MM demonstrated that *PCDH10* could down-regulate the IKKs expression and subsequently reduce the phosphorylated IκBα, leading to the blockage of p65 translocation to nucleus [19]. Meanwhile, the NF-κB constitutional activation were also found in DLBCL, especially the ABC subtype. Thus, NF-κB pathway was speculated that may drive the *PCDH10*-introduced tumorigenesis in DLBCL.

## Conclusions

This study reveals that 54.2% of DLBCL harbored *PCDH10* promoter methylation. The frequency was much higher than that of RL/FH, chronic tonsillitis and Castleman diseases. Patients with methylated *PCDH10* performs more aggressive OS and PFS. Thus, we conclude that *PCDH10* methylation status could serve as a valuable prognostic indicator, and a potential therapeutic target for demethylating drugs in the future.

## Additional files

Additional file 1: Table S1. The clinicopathological characteristics and PCDH10 methylation status of 107 cases are showed in this table. (XLS 32 kb)

Additional file 2: Table S2. The details of survival (OS and PFS) and other clinicopathological characteristics of 65 RCHOP-treated cases are showed in this table. (XLS 40 kb)

Additional file 3: Diagram S1. Description: The location of the primers in relation to the PCDH10 promoter and start site. (PPTX 194 kb)

## Abbreviations

COO: Cell of origin
DLBCL: Diffuse large B-cell lymphoma
FFPE: Formalin-fixed, paraffin-embedded
HR: Hazard ratio
IPI: International Prognostic Index
MM: multiple myeloma
MSP: Methylation-specific PCR
OS: Overall survival
PFS: Progress-free survival
RL/FH: Reactive lymph node/follicular hyperplasia
