# Supplementary material for: Clinical significance of PCDH10 promoter methylation in diffuse large B-cell lymphoma
Source: BMC Cancer. 2017 Dec 4;17:815. doi: 10.1186/s12885-017-3810-7 (PMC5715993; doi:10.1186/s12885-017-3810-7)
Supplement: Supplementary file 3 — Description: The location of the primers in relation to the PCDH10 promoter and start site. (PPTX 194 kb) [file 12885_2017_3810_MOESM3_ESM.pptx]

## Slide 1
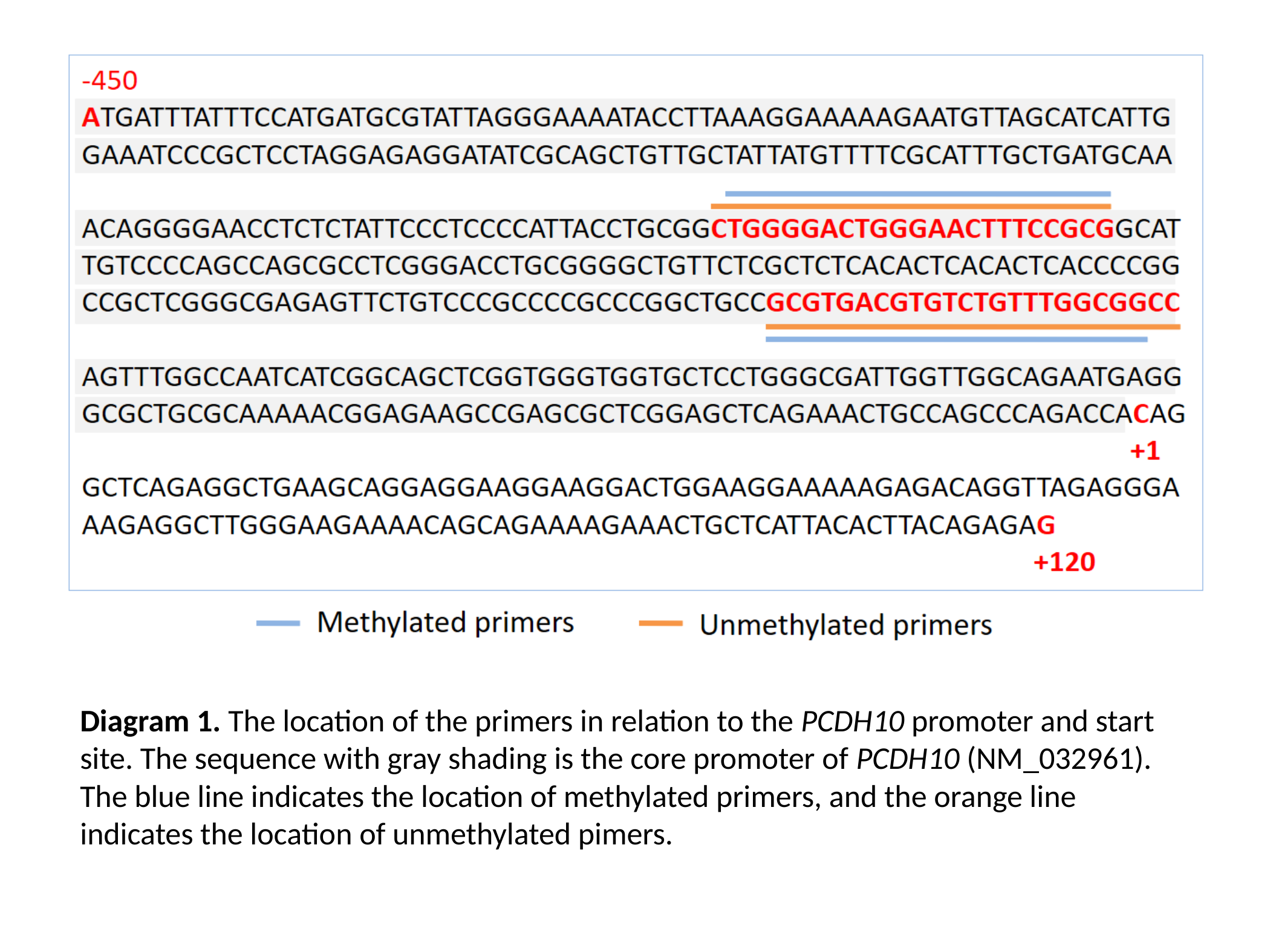

Diagram 1. The location of the primers in relation to the PCDH10 promoter and start site. The sequence with gray shading is the core promoter of PCDH10 (NM_032961). The blue line indicates the location of methylated primers, and the orange line indicates the location of unmethylated pimers.
